# The LiaFSR and BsrXRS Systems Contribute to Bile Salt Resistance in *Enterococcus faecium* Isolates

**DOI:** 10.3389/fmicb.2019.01048

**Published:** 2019-05-10

**Authors:** Luoxiong Zhou, Lihong Wang, Ping Tian, Tingting Bao, Lianbin Li, Xin Zhao

**Affiliations:** ^1^College of Animal Science and Technology, Northwest A&F University, Xianyang, China; ^2^Department of Animal Science, McGill University, Montreal, QC, Canada

**Keywords:** two-component systems, *Enterococcus faecium*, bile salts, LiaFSR, BsrXRS

## Abstract

Two-component systems (TCSs) are dominant regulating components in bacteria for responding to environmental stimuli. However, little information is available on how TCSs in *Enterococcus faecium* respond to bile salts – an important environmental stimulus for intestinal bacteria. In this study, the gene expression of 2 TCSs, BsrXRS and LiaFSR, was positively correlated with survival rates of different *E. faecium* isolates during exposure to ox gall. Moreover, gene disruptions of *bsrR*, *bsrS*, *liaS*, and *liaR* significantly reduced the survival rates of *E. faecium* in the presence of ox gall. Finally, EMSA results indicated that BsrR functioned as a transcription regulator for expression of its own gene as well as lipoate-protein ligase A (*lplA*). Additional 27 potential target genes by BsrR were revealed through *in silico* analyses. These findings suggest that BsrXRS and LiaFSR systems play important roles in bile salt resistance in *E. faecium*.

## Introduction

Bile salts are known as a natural detergent that emulsifies and solubilizes lipids, thereby playing a crucial role in fat digestion and absorption. At the same time, the amphipathic property of bile salts may induce membrane damage and impair membrane functions of intestinal bacteria. In addition to attacking cell membrane, bile salts have numerous other toxic effects on bacterial cells including disturbing macromolecule stability and inducing oxidative stress, DNA damage and protein misfolding in bacterial cells (Ruiz et al., 2013). Thus, the ability to adapt and respond to bile salts is essential for the survival and persistence of intestinal bacteria in gastrointestinal tracts.

Two-component systems (TCSs) are a primary means by which bacteria sense and respond to their environmental changes (Capra and Laub, 2012; Groisman, 2016). Although exact mechanisms for bacteria to respond to bile salts have not been completely elucidated, it is likely to be associated with TCSs. A typical TCS consists of two proteins: a signal-sensing histidine protein kinase (HK) in the cell membrane that senses an environmental change, and a cytoplasmic cognate response regulator (RR) that generates an appropriate response, usually regulating expression of target genes.

Several studies have shown that several types of bacteria can up-regulate expression of TCS genes and proteins in the presence of bile salts. For example, exposure to bile salts significantly increased expression of *baeS-baeR*, *phoP3-phoR3*, and *vraR-vraS* in *Lactobacillus rhamnosus* GG (Koskenniemi et al., 2011). A whole-genome microarray analysis of *Lactobacillus acidophilus* NCFM found a significant up-regulation of a TCS operon (LBA1425 to LBA1432) in response to bile salts and mutations in the HK (LBA1430) and the RR (LBA1431) of this operon decreased the survival rates (Pfeiler et al., 2007). Similarly, *basR* and *basS* genes were up-regulated after exposure to bile salts in *Escherichia coli* O157:H7 (Kus et al., 2011). *E. coli* Nissle 1917 became more resistant to bile salts after introduction of a mutated *envZ* (*envZ*_P41L_) from *E. coli MG1655* (the *envZ* gene encodes a histidine kinase and induces a higher level of phosphorylated OmpR) (Adediran et al., 2014). In *Propionibacterium freudenreichii* SI41, addition of bile salts was found to induce expression of a putative RR (ORF0001) protein at two-dimensional electrophoresis (Leverrier et al., 2003). In another study, a RR (BL1000) was detected when exposure to ox bile in *Bifidobacterium longum* NCIMB 8809 (Sanchez et al., 2005). Disruption of *cbrR* in *Campylobacter jejuni* F38011 rendered the strain more sensitive to ox bile (Raphael et al., 2005). Mutation of the *cpxAR* in *Klebsiella pneumoniae* NTUH-K2044 led to low survival rates during exposure to bile at the concentrations higher than 0.5% (Srinivasan et al., 2012). Le Breton et al. (2003) found that mRNA amount of *ehk*10-*err*10 was increased after incubation with 0.08% bile salts in *Enterococcus faecalis* JH2-2. However, the expression of *ehk*10-*err*10 TCS was not significantly increased in a transcriptional analysis of *E. faecalis* V583 response to ox gall (Solheim et al., 2007). Collectively, these findings suggest that TCSs are essential for resistance to bile salts in various bacterial species. However, there is no information available on involvement of TCSs in the bile resistance in *E. faecium*.

*Enterococcus faecium* is a common commensal organism in the gastrointestinal tract of a wide variety of hosts. At the same time, it is also an important cause of multidrug-resistant hospital-acquired infection (Lebreton et al., 2013). Moreover, *E. faecium* strains have been considered as potential bio-preservatives due to the strong antimicrobial activity against *Listeria monocytogenes* in fermented food production (Pingitore et al., 2012; Favaro et al., 2014). As an important group of lactic acid bacteria (LAB), *E. faecium* has been used as probiotics to confer health benefit to the host (Veljovic et al., 2017). *E. faecium* harbors more than 15 TCSs in the chromosome and in plasmids (Ortet et al., 2015). Four TCSs in *E. faecium*, CroRS, ChtRS, LiaFSR, and VanSR, have been characterized for antibiotic resistance. The CroRS system responds to cell wall stress and is critical for the resistance against cell wall-targeting antibiotics in *E. faecium*. Mutation of *croRS* in the *E. faecium* 1,141,733 strain remarkably reduced the resistance to cefepime and ceftriaxone (Kellogg et al., 2017). Guzman Prieto et al. (2017) revealed that the ChtRS system contributed to chlorhexidine tolerance. Mutants of *chtR* and *chtS* exhibited a slower exponential growth rate than the wild-type *E. faecium* E1162 in the presence of chlorhexidine. The LiaFSR system is an important regulator of the cell envelope stress response to the membrane-targeting agents (Tran et al., 2016). This system consists of a sensor histidine kinase (LiaS), a response regulator (LiaR) and a membrane-anchored protein (LiaF) that negatively affect the function of LiaS. The role of LiaFSR in enterococci has been described recently in resistance to daptomycin (Tran et al., 2016). Deletion of *liaR* in *E. faecium* R446F and R497 that resistant to daptomycin fully reversed resistance (Panesso et al., 2015). The VanSR two-component system regulates the downstream *van* gene cluster, which alters the cell wall precursors to allow bacteria to resist vancomycin (Hong et al., 2008).

The objective of this study was to identify potential TCSs in the resistance of *E. faecium* to bile salts. Our results show that the gene expression of *liaS*, *liaR*, *bsrR*, and *bsrS* was positively correlated with survival rate of different *E. faecium* isolates after exposure to bile salts. Moreover, gene disruptions of *bsrR*, *bsrS*, *liaS*, and *liaR* significantly reduced the survival rates of *E. faecium* after exposure to different concentrations of bile salts. In addition, BsrR could function as a transcription regulator for its own expression and expression of *lplA.* The *in silico* analyses also revealed 27 other target genes for BsrR. These findings provide a first glance at the molecular mechanisms for bile salt resistance in *E. faecium*.

## Materials and Methods

### Bacterial Strains, Plasmids, and Culture Conditions

*Enterococcus faecium* isolates, *E. coli* strains and plasmids used in this study are listed in Table 1. *E. faecium* were isolated from a previous study (Feng et al., 2016). *E. faecium* isolates were identified by 16s rRNA gene sequencing and subsequent blasting of the sequences, using the Basic Local Alignment Search Tool (BLAST) program. The genomic DNA was extracted from overnight culture using an EasyPure Genomic DNA kit (TransGen Biotech, Beijing, China) as per manufacturer’s protocol. A PCR reaction was performed to amplify the 16S rRNA genes using universal primers 27F and 1492R (Supplementary Table S1). PCR products were then sequenced using the Sanger sequencing. *E. faecium* isolates were routinely grown in the de Man, Rogosa, and Sharpe (MRS) broth or on the agar at 37°C. *E. coli* EC1000 (Leenhouts, 1996) was used for plasmid construction and was grown in the Luria-Bertani (LB) broth at 30°C. Where necessary, antibiotics were used at the following concentrations: chloramphenicol 5 μg/ml for *E. faecium*, spectinomycin 300 μg/ml for *E. faecium*, and 100 μg/ml for *E. coli*, ampicillin 100 μg/ml for *E. coli*. All antibiotics were obtained from Sigma (St. Louis, MO, United States).

**TABLE 1:** Strains and plasmids used in this study.

**Strain or**		
**plasmid**	**Description^b^**	**References**
**Strains**		
*E. faecium*^a^		
NW1	Isolated from duodenum	Feng et al.,2016
NW2	Isolated from duodenum	Feng et al.,2016
NW3	Isolated from duodenum	Feng et al.,2016
NW4	Isolated from cecum	Feng et al.,2016
NW5	Isolated from muscular stomach	Feng et al.,2016
NW6	Isolated from cecum	Feng et al.,2016
NW7	Isolated from muscular stomach	Feng et al.,2016
NW8	Isolated from jejunum	Feng et al.,2016
*liaR*::pWS3	Single-crossover insertional mutant of *liaR* of NW2	This study
*liaS*::pWS3	Single-crossover insertional mutant of *liaS* of NW2	This study
*bsrR*::pWS3	Single-crossover insertional mutant of *bsrR* of NW2	This study
*bsrS*::pWS3	Single-crossover insertional mutant of *bsrS* of NW2	This study
*liaS*::pWS3+*liaS*	Complementation strain of *liaS*::pWS3; *liaS*::pWS3 harboring pRB473*-liaS*	This study
*E. coli*		
EC1000	Host strain for pWS3 derived vectors	Leenhouts,1996
DH5α	Host strain for routine cloning	Lab stock
BL21 (DE3)	*E. coli* overexpression host	Lab stock
**Plasmids**		
pWS3	Gram-positive thermosensitive plasmid for gene disruption, Spec^r^	Zhang et al.,2011
pWS3-LiaR	pWS3 containing a 489-bp KpnI/EcoRI internal fragment of *liaR* gene	This study
pWS3-LiaS	pWS3 containing a 827-bp KpnI/EcoRI internal fragment of *liaS* gene	This Study
pWS3-BsrR	pWS3 containing a 505-bp KpnI/EcoRI internal fragment of *bsrR* gene	This Study
pWS3-BsrS	pWS3 containing a 1,015-bp KpnI/XhoI internal fragment of *bsrS* gene	This Study
pRB473	Broad-range shuttle vector, Amp^r^, Cm^r^	Brückner et al.,1993
pRB473*-liaS*	Complementation plasmid for *liaS*, pRB473 carrying gene *liaS*	This Study
pET21b (+)	The expression plasmid, Amp^r^	Lab stock
pET21b-BsrR	The expression plasmid for BsrR protein	This Study

**^a^All E. faecium isolates were isolated from the chicken gastrointestinal tract.^b^Spec^r^, spectinomycin resistance; Amp^r^, Ampicillin resistance; Cm^r^, chloramphenicol resistance*.*

### Resistance of *E. faecium* to Bile Salts

To determine the resistant abilities of 8 *E. faecium* isolates to bile salts, overnight cultures were diluted 100-fold in 20 ml of the fresh MSR medium. When bacterial cells were grown to the mid-log-phase (OD_600_ of ∼0.4), 1 ml aliquots of the cultures were collected as control samples (*t* = 0 min). Another 1 ml aliquots of the cultures were collected and centrifuged at 12,000 × *g* for 2 min, then re-suspended in the same volume of the fresh medium supplemented with different concentrations of 0.5, 1, 2, and 5% bile salts (Ox gall, Sigma, United States). After incubation for 30 min at 37°C (*t* = 30 min), the numbers of colony-forming units per milliliter (CFU/ml) of each sample were determined by plating a serial of 10-fold dilutions on MRS agar and then incubated at 37°C for 48 h. The percentage survival of *E. faecium* in bile salts was calculated by comparing CFU at 30 min to CFU at time zero. The assays were performed in three independent experiments.

### RNA Preparation, RT-PCR, and RT-qPCR

Eight TCSs in *E. faecium* genomes were selected to study the effect of TCSs on resistance to bile salts. Among them, 5 TCSs are homologous to TCSs in other species known to be involved in bile resistance (Supplementary Table S2), while ChtRS, CroRS, and LiaFSR were selected because they respond to cell envelope-targeting antimicrobial agents. To quantify gene expression, overnight cultures were diluted 100-fold in 20 ml of the fresh MSR medium. When bacterial cells were grown to the mid-log-phase (OD_600_ of ∼0.4), 5 ml aliquots of the culture were centrifuged for 2 min at 12,000 × *g* at 4°C and pellets were flash frozen in liquid N2 prior to RNA extraction. This sample served as the control (*t* = 0 min) prior to the addition of bile salts. Bile salts (final concentration 0.5%, w/v) were added into the remaining culture. After 30 min of incubation at 37°C, 5 ml aliquots of the culture were centrifuged and flash frozen as described above. Total RNA was extracted by using the E.Z.N.A. Bacterial RNA Kit (Omega, United States). One μg of extracted RNA was converted into cDNA using a PrimeScript^TM^ RT reagent Kit (Takara, Dalian, China) according to the manufacturer’s instructions. To identify the transcript structure, reverse transcription-PCR (RT-PCR) was performed on cDNA synthesized from wild-type *E. faecium* NW2 RNA with primers listed in Supplementary Table S1. RNA without RT and genomic DNA were used as the negative control and positive control, respectively. The levels of expression of genes were determined by quantitative real-time RT-PCR (RT-qPCR) using a real-time PCR kit (Takara, Dalian, China) and specific primers (Supplementary Table S1). The transcript levels were determined by the 2^–ΔΔCt^ method using the *adk* gene as an endogenous control gene.

### Generation of Insertional Mutants and *in trans* Complementation

To verify whether the LiaFSR and BsrXRS systems were responsible for resistance to bile salts, gene disruption mutants were constructed in *E. faecium* NW2 as previously described Zhang et al. (2011). The internal DNA fragments of *liaR*, *liaS*, *bsrR*, and *bsrS* genes were amplified using primers LiaR-mut-F/LiaR-mut-R, LiaS-mut-F/LiaS-mut-R, BsrR-mut-F/BsrR-mut-R, and BsrS-mut-F/BsrS-mut-R, respectively (Supplementary Table S1), and cloned to a Gram-positive thermosensitive vector pWS3. The resulting constructs were transformed into *E. coli* EC1000 for propagation and grown on LB plates containing 100 μg/ml spectinomycin at 30°C. The inserts from each construct were sequenced using the primers M13-F and SK-R to ensure that no mutations arose during cloning. The correct vectors (Table 1) were then electrotransformed into *E. faecium* NW2 using Gene Pulser Xcell^TM^ apparatus (Bio-Rad), operating at 2,500 V, 25 mF capacitance, 200 Ω resistance, and 2-mm cuvettes that contained 2 μg of plasmid DNA plus 100 μl of electrocompetent cells. The electrocompetent cells were obtained as previously described (Zhang et al., 2012). After electrotransformation, the cells were allowed to recover for 2 h at the permissive temperature of 30^∘^C, after which the cells were plated on BHI plates supplemented with 300 μg/ml spectinomycin at 30°C to select for transformants. Spectinomycin-resistant colonies were picked and grown overnight in 2 ml of BHI broth supplemented with 300 μg/ml spectinomycin at an elevated temperature (37°C) to cure the plasmid. Following serial passages at 37°C, the cells were then plated on BHI agar plates with 300 μg/ml spectinomycin at 37°C. Single-cross-over integration into *liaR*, *liaS*, *bsrR*, or *bsrS* was verified by PCR with pWS3-specific primer SK-R and gene-specific primers LiaR-d-check, LiaS-d-check, BsrR-d-check, BsrS-d-check, respectively (Supplementary Table S1).

The *liaS* mutant was complemented *in trans* by cloning *liaS* gene into the shuttle plasmid pRB473 (Brückner et al., 1993). The gene *lias* fragment was generated from the NW2 genomic DNA using primers LiaS-comp-F and LiaS-comp-R (Supplementary Table S1). The PCR product was then ligated into pRB473, generating the complementation plasmid pRB473-*liaS*. Sequencing of the full insert was performed to verify the absence of errors. The correct plasmid was introduced into the *liaS* mutant strain by electroporation as described above. Unfortunately, despite repeated attempts, we were unable to obtain complemented strains for *liaR*, *bsrR*, and *bsrS* genes.

### Search of the Binding Sites and Target Genes of BsrR in *E. faecium in silico*

Available complete genome sequence of the *E. faecium* DO was downloaded from the NCBI RefSeq database in GenBank (.gbk) format. The amino acid sequence of BsrR orthologs from 7 bacterial species (including *E. faecium* DO) in the *Enterococcus* genus were downloaded from OrtholugeDB (Whiteside et al., 2013). The upstream DNA sequences of BsrXRS system from 7 bacterial species were obtained from the NCBI GenBank database. Then these DNA sequences were used to search the putative binding sites for BsrR using the motif finding in DMINDA 2.0 web server with default parameters (Yang et al., 2017). As for searching target genes, all promoter sequences in the *E. faecium* DO genome were downloaded from DOOR 2.0 (Mao et al., 2014) and submitted to DMINDA 2.0. Multiple sequence alignments of DNA and protein sequences were performed using the CLUSTAL W (Thompson et al., 1994).

### Overexpression and Purification of BsrR

To further examine if the predicted binding sites are directly bound by BsrR, we used the electrophoretic mobility shift assays (EMSAs) to detect the protein–DNA interactions. The *bsrR* gene was amplified from the NW2 genome using primers BsrRc-F/BsrRc-R and cloned into the pET21b vector, then was transformed into *E. coli* strain BL21 (DE3) for overexpressing C-terminally six-His-tagged BsrR. Successful transformants were inoculated into LB broth containing ampicillin (100 μg/ml) and incubated at 37°C with vigorous shaking (200 rpm) to achieve an OD_600_ of 0.55. Once the correct OD_600_ obtained, cultures were kept on ice for 10 min followed by the addition of 0.1 mM (final concentration) isopropyl-β-d-1-thiogalactopyranoside (IPTG) to induce BsrR expression. The cultures were further incubated at 18°C for overnight, and the cells were harvested by centrifugation at 12,000 × *g* for 5 min at 4°C. To purify the recombinant BsrR, the cell pellet was resuspended in binding buffer [15 mM imidazole, 50 mM Tris-HCl, 0.5 mM EDTA, 50 mM NaCl and 10% glycerol (v/v), pH = 7.5] and disrupted by sonication, and the cell debris was removed by centrifugation at 12,000 × *g* for 15 min at 4°C. This soluble fraction, which contained the BsrR protein, was placed in a HisTrap prepacked column (GE healthcare). The same binding buffer was used for equilibration of the column. Whole-cell lysate was allowed to bind to the resin. The column was then washed five times with binding buffer. Bound BsrR were eluted using elution buffer containing 250 mM imidazole [250 mM imidazole, 50 mM Tris-HCl, 0.5 mM EDTA, 50 mM NaCl, 10% glycerol (v/v), pH = 7.5]. The eluted samples were dialyzed in the storage buffer [10 mM Tris, 50 mM KCl, 1 mM DTT, 10% glycerol (v/v), pH 7.5] at 4°C. The concentration of BsrR protein was determined by the Bradford assay (Bio-Rad) using the bovine serum albumin (BSA) as the standard.

### Electrophoretic Mobility Shift Assays (EMSAs)

The intergenic regions upstream of *bsrX* (143 bp) and *lplA* (162 bp) were amplified from the *E. faecium* NW2 genomic DNA using the primers pBsrRS-F/pBsrRS-R and pLplA-F/pLplA-R (Supplementary Table S1), respectively. A DNA fragment as a negative control was amplified from the coding region of housekeeping gene *ddlA* using the primer pNC-F/pNC-R, which does not share sequence identity with the upstream of *bsrX* and *lplA*. The EMSA experiments were performed using the LightShift EMSA Optimization and Control Kit according to the manufacturer’s instructions (Pierce, Thermo Fisher Scientific, Waltham, MA, United States). Briefly, the 5′-end biotin labeled DNA probes (20 fmol) were incubated with various concentrations of recombinant BsrR protein (0, 0.1, 0.2, and 0.3 nM) in binding reaction (20 μl) containing 10 mM Tris-HCl (pH 7.5), 50 mM KCl, 0.5 mM DTT, 1 mM EDTA, 7.5% glycerol, 1 mg of BSA per ml, and 1 μg of poly (dI-dC). To verify the specificity of BsrR-probe interaction, 200-fold excess amounts of unlabeled non-specific DNA (negative control) or each unlabeled specific probe were first incubated with BsrR protein for 20 min, followed by the addition of each labeled specific probe. Reactions were incubated for 40 min at 25°C, and then were loaded in 6% non-denaturing polyacrylamide gels containing 5% glycerol and electrophoresed in the 0.5 × Tris-borate-EDTA (TBE) for 90 min at 120 V and 4°C. Biotin-labeled DNA-protein complexes were detected using the Chemiluminescent Nucleic Acid Detection Module Kit follow according to the manufacturer’s instructions (Pierce, Thermo Fisher Scientific).

### Statistical Analysis

One-way analysis of variance (ANOVA) was used to analyze data using the R software (Version 3.4.3) (R Core Team, 2017). The differences between treatment means were calculated using the Duncan’s new multiple range test in the Agricolae Packages (Version 1.2-8) (Mendiburu, 2017). Data are expressed as mean ± standard deviation (SD) and *P* < 0.05 was considered significantly different.

## Results

### Resistance of *E. faecium* to Ox Gall

**FIGURE 1:**
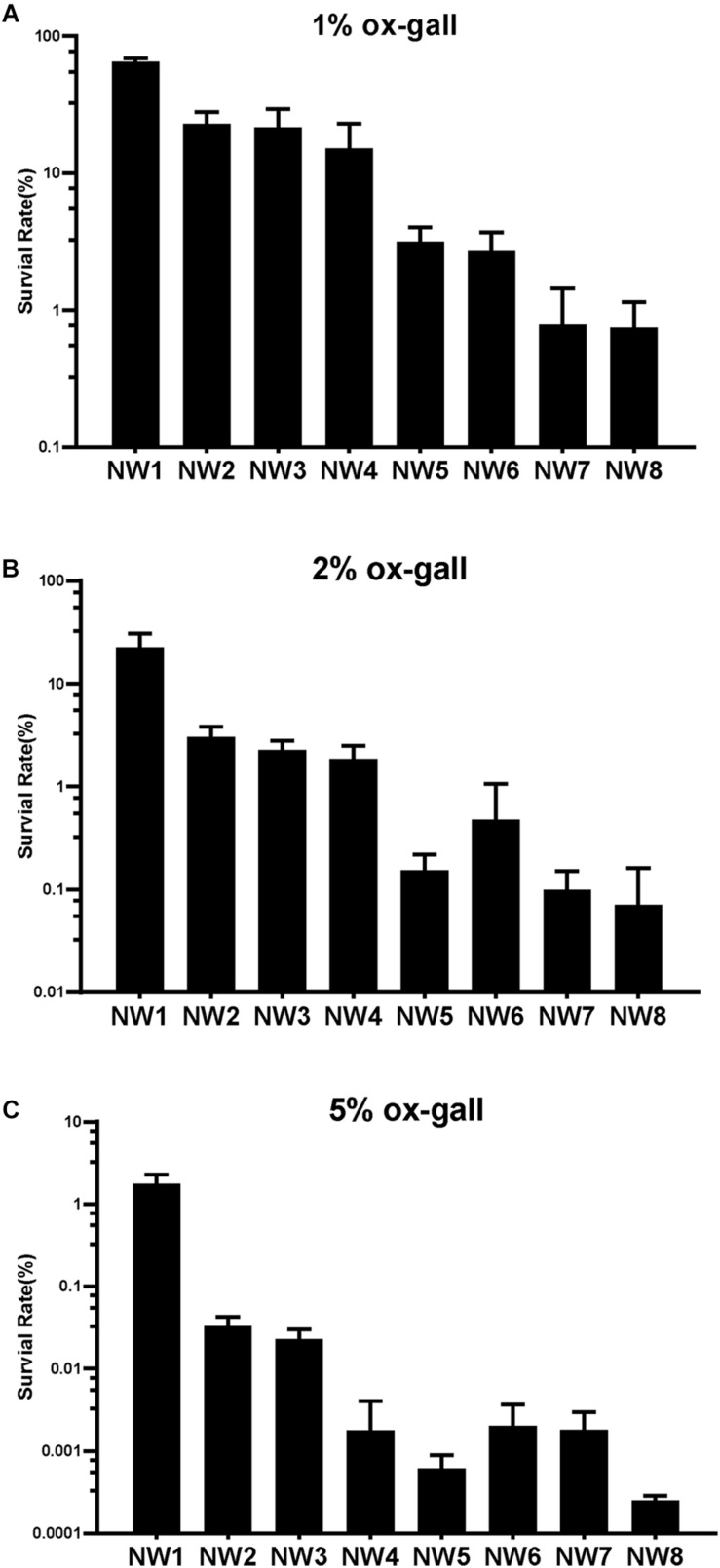
Resistance of *Enterococcus faecium* to bile salts. Survival rates of *E. faecium* isolates after 30 min exposure to 1 **(A)**, 2 **(B)**, and 5% **(C)** ox gall. Data are shown as mean ± SD from three independent biological replicates.

**FIGURE 2:**
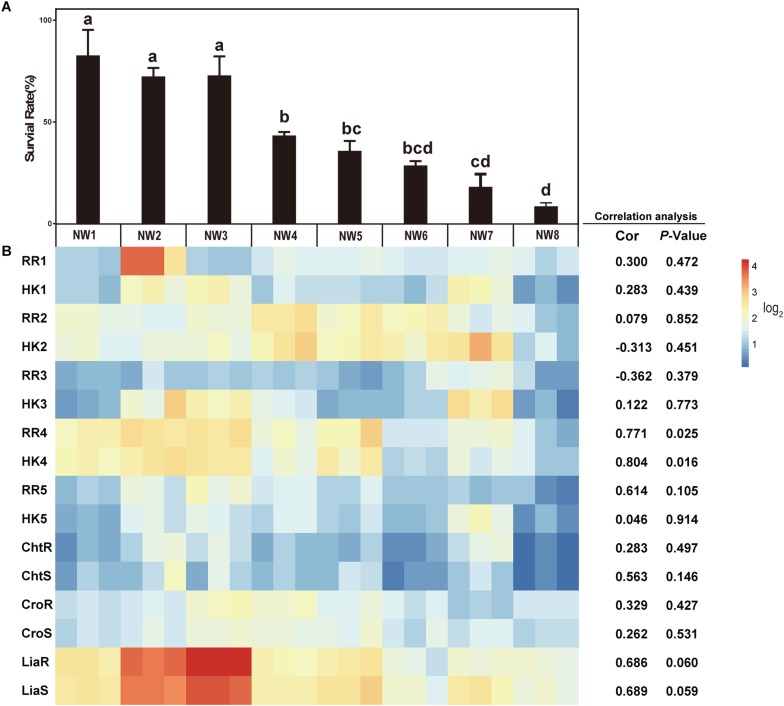
The correlations between the gene expressions of TCSs and bile salt resistance. **(A)** Survival rates of *E. faecium* isolates after 30 min exposure to 0.5% ox gall. Data are shown as mean ± SD from three independent biological replicates. Different letters above bars indicate the survival rates are significant differences among *E. faecium* isolates (*P* < 0.05). **(B)** The heatmap of expression of TCS genes after 0.5% ox gall addition compared with the time point preceding the bile addition (**B**, left panel). Red indicates up-regulated gene expression and blue denotes down-regulated gene expression. The color bar right indicates the expression scales [represented as log_2_ (fold change)]. Correlations between the survival rates in 0.5% ox gall and the expressions of TCS genes in *E. faecium* were performed using Pearson’s product-moment correlation in R software. The Cor value represents the correlation coefficient and *P*-value < 0.05 indicates that the correlation is significantly different.

To assay the resistant abilities to ox gall of 8 *E. faecium* isolates, the survival rates were measured in the MRS broth supplemented with different concentrations of ox gall. The resistant activities to bile salts varied widely among the isolates. After exposure to 1% ox gall, more than 20% of *E. faecium* NW1, 2, and 3 remained viable, whereas the survival rate of NW7 and 8 fell below 1% (Figure 1A). After exposure to 2% ox gall, *E. faecium* NW1, 2, 3 still had more than 1% survival rates, whereas survival rates of *E. faecium* NW7 and 8 were lower than 0.1% (Figure 1B). When the concentration of ox gall was increased to 5%, the survival of all *E. faecium* isolates except NW1 was less than 0.1% (Figure 1C). On the other hand, three isolates (*E. faecium* NW1, 2, and 3) showed more than 70% survival rates after exposure to 0.5% ox gall (Figure 2A). The 0.5% ox gall was selected for further studies, unless indicated otherwise.

### The Correlations Between the Gene Expression of Putative TCSs and Bile Salt Resistance

To determine whether TCSs were involved in the resistance of *E. faecium* to bile salts, 8 TCSs were selected for measuring the expression of these genes in response to 0.5% ox gall. As shown in Figure 2B, there was a significantly positive correlation between the survival rate and the expressions of *RR4* (0.771, *P* = 0.025) and *HK4* (0.804, *P* = 0.016). The positive correlation between the survival rate and the expressions of *liaR* (0.686, *P* = 0.060) and *liaS* (0.689, *P* = 0.059) tended to be significant. These results suggested that *liaR*, *liaS*, *RR4*, and *HK4* genes may contribute to bile salt resistance in *E. faecium*. The correlations between the gene expression of the remaining putative or known TCSs and bile salt resistance were not significant.

### The Effect of *liaR*, *liaS*, *bsrR*, and *bsrS* Mutants on the Bile Salt Resistance in *E. faecium* NW2

Considering the importance of *RR4* and *HK4* genes in bile salt resistance, these putative and previously unstudied genes were renamed as *bsrR* and *bsrS*, for b ile s alt r esistance r esponse regulator and b ile s alt r esistance s ensor histidine kinase, respectively.

**FIGURE 3:**
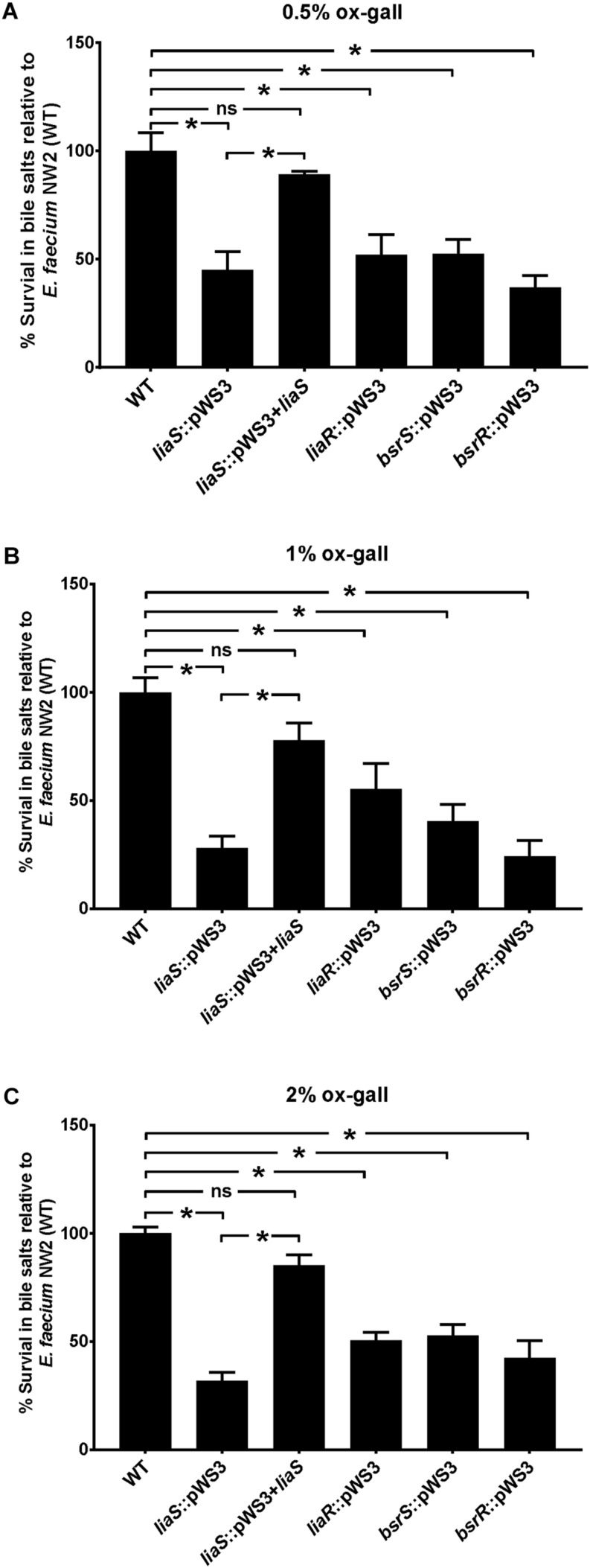
The effect of mutation of *liaR*, *liaS*, *bsrR*, and *bsrS* genes on resistance to ox gall by *E. faecium* NW2. Survival rates of *E. faecium* NW2 wild type (WT) and its mutants with insertional mutagenesis of *liaS* (*liaS:*:pWS3), *liaR* (*liaR*::pWS3), *bsrR* (*bsrR*::pWS3), and *bsrS* (*bsrS*::pWS3) genes and the complementation strain of *liaS* (*liaS:*:pWS3 + *liaS*) after 30 min exposure to 0.5 **(A)**, 1 **(B)**, 2% ox gall **(C)**. Data are mean ± SD of three independent experiments. Asterisk above bars indicate the survival rates are significant differences between WT and mutant strains or complementation strain (*P* < 0.05).

To confirm the involvement of *liaR*, *liaS*, *bsrR*, and *bsrS* genes in resistance to bile salts, mutants of gene disruption were constructed using *E. faecium* NW2 due to its high bile resistant ability and up-regulation of *liaR*, *liaS*, *bsrR*, and *bsrS* genes after exposure to ox gall. All mutants had similar growth curves as the parental strain NW2 in MRS broth in the absence of ox gall (data not shown). As showed in Figure 3, disruption of *liaR*, *liaS*, *bsrR*, and *bsrS* significantly decreased the survival rate after exposure to 0.5, 1, and 2% ox gall compared to the *E. faecium* NW2 wild type. Complementation of the *liaS* gene resulted in restoration of survival in ox gall to levels of wild-type NW2 (Figure 3). However, the *liaR*, *bsrR*, and *bsrS* complemented strains were not obtained after several attempts. These results indicated that *liaR*, *liaS*, *bsrR*, and *bsrS* were important for bile salt resistance in *E. faecium*.

### Characterization of the BsrXRS System in *E. faecium*

**FIGURE 4:**
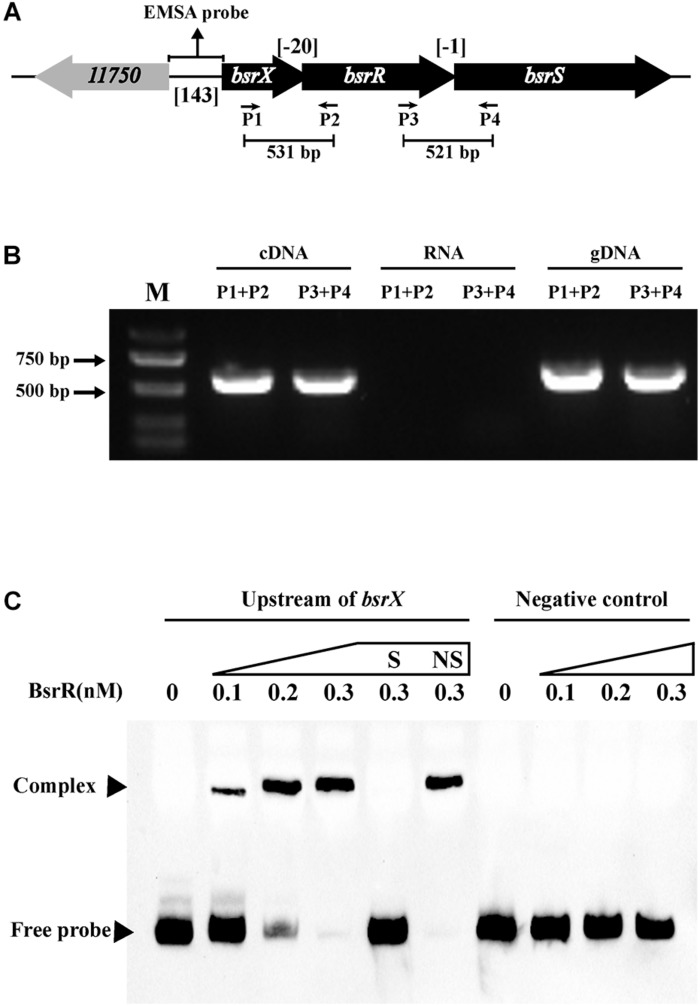
Characterization of the BsrXRS system in *E. faecium*. **(A)** Schematic representation of the BsrXRS system in *E. faecium*. **(B)** RT-PCR was performed for studying co-transcription using cDNA, RNA (negative control) and genomic DNA (positive control). Location of each intergenic primer pair for RT-PCR is shown in **(A)**. **(C)** EMSA using biotin-labeled probes containing the intergenic region upstream of *bsrX* (143-bp) or negative control (150 bp) incubated with indicated amounts of BsrR protein. The negative control was amplified from the coding region of housekeeping gene *ddlA*, which does not share sequence identity with the intergenic region upstream of *bsrX*. EMSAs in the presence of 200-fold unlabeled specific probe (S, upstream of *bsrX*) or non-specific (NS, negative control) competitor DNA were performed as controls. Free DNA fragments and BsrR-DNA complexes are labeled.

In analysis of the genomic location of *bsrR* and *bsrS*, it was found that the 5′-end of the *bsrR* overlaps with 20 bases of the 3′-end of a 351-bp gene encoding a hypothetical protein (HMPREF0351_11749, renamed as *bsrX*) and the 5′-end of the *bsrS* overlaps with a base of the 3′-end of *bsrR*, which suggest that *bsrX*, *bsrR*, and *bsrS* may exist in an operon (Figure 4A). We validated that these 3 genes were co-transcribed, forming a single operon by RT-PCR (Figure 4B).

The sequence analysis of BsrR revealed that the C-terminal of BsrR harbors a DNA-binding domain, suggesting that BsrR may function as a transcription regulator. To examine whether BsrR autoregulates directly, we performed EMSA with purified BsrR protein. The BsrR was showed to bind to a 143-bp intergenic region directly upstream of *bsrX*, thus suggesting BsrR to directly regulate the transcription of its own operon (Figure 4C).

To further determine whether BsrR in the *Enterococcus* genus can function as a transcription regulator, protein sequences of the BsrR from 7 strains including *E. faecium* DO were aligned. There were 86% identities and a conserved DNA-binding domain (Thr-164, Ser-183, Arg-184, Thr-202, Ala-205, Lys-213, Ile-214, Thr-224, and Gly-229) (Supplementary Figure S1), implying that these BsrR homologs might have a conserved DNA-binding site. By submitting the DNA sequences of upstream of the BsrXRS system from 7 bacterial species to the DMINDA 2.0 web server (Yang et al., 2017), a putative BsrR-binding site, the consensus sequence TMGAGTATAMTA, was found in the *Enterococcus* genus bacteria (Table 2).

**TABLE 2:** Putative BsrR-binding sites in the promoter of the BsrXRS system.

		**Distance^a^**	**(bp)**	
**Strain**	**Site**	***d*_TLS_**	***d*_–__10_**	**Locus Tag**
*E. faecium* DO	TAGAGTATACTA	−38	−5	HMPREF0351_11749
*E. mundtii* QU 25	TAGAGTATACTA	−37	−5	EMQU_1779
*E. hirae* ATCC 9790	TAGAGTATACTA	−37	−5	EHR_09675
*E. faecalis* V583	TAGAGTATACTA	−54	−5	EF1260
*E. gallinarum* FDAARGOS_163	TCGAGTATACTA	−33	−5	AL523_09775
*E. casseliflavus* EC20	TCGAGTATACTA	−33	−5	ECBG_02917
*E. silesiacus* LMG 23085	TAGAGTATAATA	−51	−5	ATZ33_03865

**^a^The d_TLS_ and d_–__10_ columns indicate the distance from the 5′ end of the site to the 5′ end of the predicted translation start site (TLS) and the −10 promoter element, respectively*.*

### Characterization of the Potential Target Genes for the BsrXRS System

To determine whether BsrR in the *Enterococcus* genus can also function as a transcription regulator for expression of other genes, an *in silico* genome-wide search was performed for putative BsrR target genes using the identified consensus sequence TMGAGTATAMTA as the BsrR-binding motif. As shown in Table 3, 27 putative target genes were identified.

**TABLE 3:** Putative target genes of BsrR in genome of *E. faecium* DO.

			**COG**
			**functional**
**Operon**	**Locus Tag**	**Product**	**categories*^a^***
1107280	HMPREF0351_11747	Sensor histidine kinase	T
	HMPREF0351_11748	Response regulator	T
	HMPREF0351_11749	Hypothetical protein	S
3824662	HMPREF0351_11750	HAD superfamily hydrolase	S
3824769	HMPREF0351_12076	Hypothetical protein	S
1107358	HMPREF0351_12077	Lipoate–protein ligase A	H
	HMPREF0351_12078	HD family metal-dependent phosphohydrolase	S
	HMPREF0351_12079	HAD superfamily hydrolase	S
3824741	HMPREF0351_11989	Thioredoxin-disulfide reductase	O
3824782	HMPREF0351_12122	GNAT family acetyltransferase	K
3824379	HMPREF0351_10965	2-hydroxy-3-oxopropionate reductase	I
3824637	HMPREF0351_11678	DNA-directed DNA polymerase III subunit alpha	L
1106980	HMPREF0351_10295	2-dehydropantoate 2-reductase	H
	HMPREF0351_10296	Regulatory protein	S
1106982	HMPREF0351_10301	Acetyltransferase	K
	HMPREF0351_10302	Hypothetical protein	K
3824725	HMPREF0351_11918	*N*-acetyltransferase GCN5	K
3824372	HMPREF0351_10954	GNAT family acetyltransferase	Q
3824373	HMPREF0351_10955	Permease	P
3824469	HMPREF0351_11172	Fosfomycin resistance protein FosX	S
3824470	HMPREF0351_11173	Hypothetical protein	S
3824507	HMPREF0351_11295	Enoyl- acyl-carrier-protein reductase NADH	I
3824508	HMPREF0351_11296	3-hydroxydecanoyl-ACP dehydratase	I
3824962	HMPREF0351_12672	Hypothetical protein	S
3824963	HMPREF0351_12673	Collagen-binding MSCRAMM Scm (Fms10)	S
3824527	HMPREF0351_11330	Glycine C-acetyltransferase	H
3824364	HMPREF0351_10944	Teichoic acid glycosylation protein GtrA	S

*^a^The one-letter codes represent the following COG functional categories: H, coenzyme transport and metabolism; I, lipid transport and metabolism; K, transcription; L, replication, recombination and repair; O, posttranslational modification, protein turnover; chaperones; P, inorganic ion transport and metabolism; Q, secondary metabolites biosynthesis, transport and catabolism; S, function unknown; T, signal transduction mechanisms.*

**FIGURE 5:**
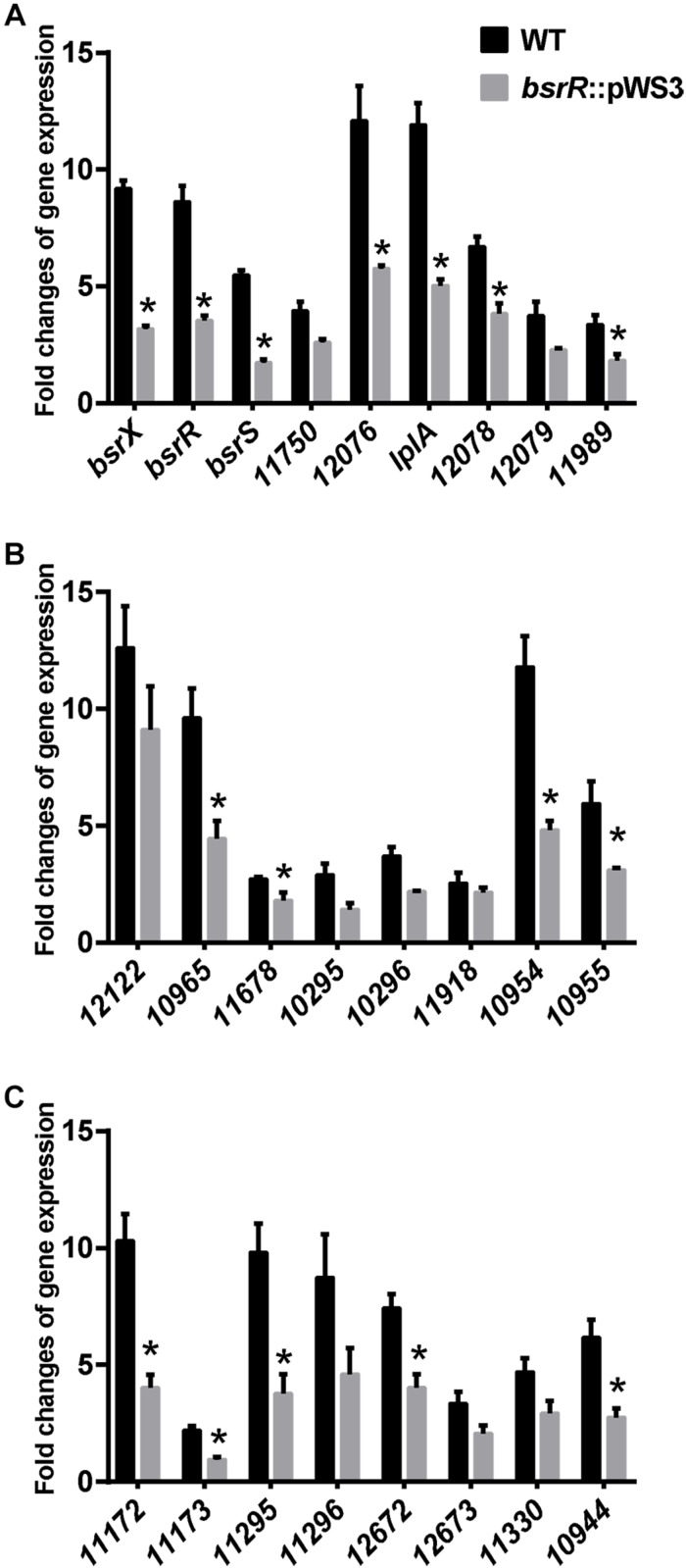
Theexpression of target genes of BsrR in response to bile salts. **(A–C)** Relative expression of target genes of BsrR in response to 0.5% ox-gall was determined by RT-qPCR. Fold changes of gene expression of wild-type (WT) and the *bsrR* mutant (*bsrR*::pWS3) of *E. faecium* NW2 after exposure to 0.5% ox-gall for 30 min, were calculated relative to WT and *bsrR*::pWS3 strains before the bile salts addition, respectively. Data are shown as mean ± SD from three independent biological replicates. Asterisk above bars indicate significant difference between WT and the *bsrR* mutant (*P* < 0.05). The target genes, *bsrX* (*HMPREF0351_11749*), *bsrR* (*HMPREF0351_11748*), *bsrS* (*HMPREF0351_11747*), 11750 (*HMPREF0351_11750*), 12076 (*HMPREF0351_12076*), *lplA* (*HMPREF0351_12077*), 12078 (*HMPREF0351_12078*), 12079 (*HMPREF0351_12079*), 11989 (*HMPREF0351_11989*), 12122 (*HMPREF0351_12122*), 10965 (*HMPREF0351_10965*), 11678 (*HMPREF0351_11678*), 10295 (*HMPREF0351_10295*), 10296 (*HMPREF0351_10296*), 11918 (*HMPREF0351_11918*), 10954 (*HMPREF0351_10954*), 10955 (*HMPREF0351_10955*), 11172 (*HMPREF0351_11172*), 11173 (*HMPREF0351_11173*), 11295 (*HMPREF0351_11295*), 11296 (*HMPREF0351_11296*), 12672 (*HMPREF0351_12672*), 12673 (*HMPREF0351_12673*), 11330 (*HMPREF0351_11330*), 10944 (*HMPREF0351_10944*), were predicted with putative BsrR-binding motif in genome of *E. faecium* DO.

**FIGURE 6:**
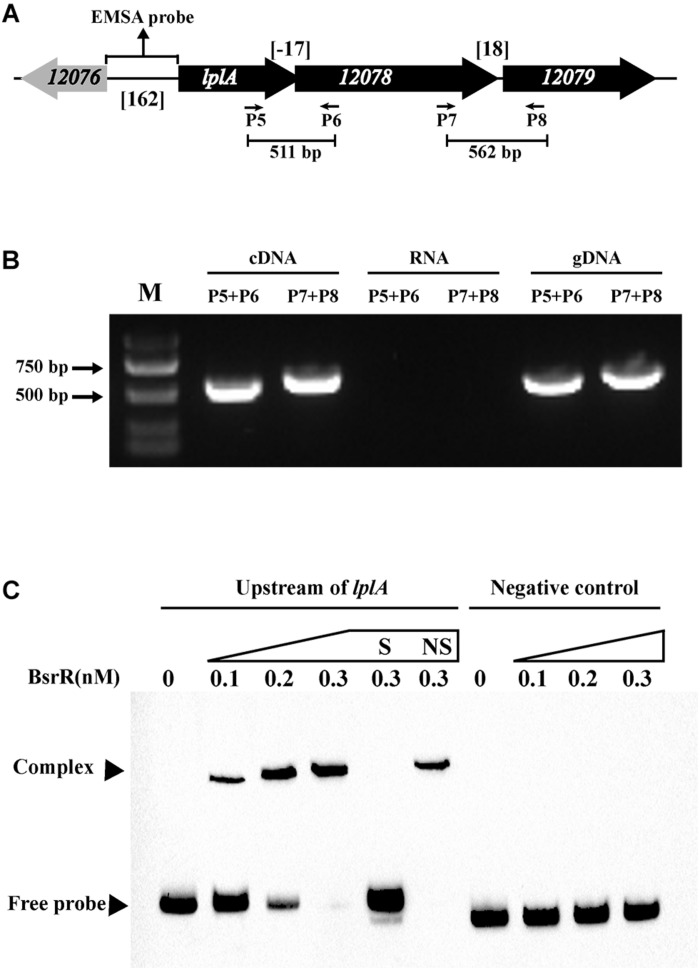
Characterization of the *lplA* operon that regulated by BsrR. **(A)** Schematic representation of *lplA* operon in *E. faecium*. **(B)** RT-PCR was performed for studying co-transcription using cDNA, RNA (negative control) and genomic DNA (positive control). Location of each intergenic primer pair for RT-PCR is shown in **(A)**. **(C)** EMSA using biotin-labeled probes containing the intergenic region upstream of *lplA* (162-bp) or negative control (150 bp) incubated with indicated amounts of BsrR protein. The negative control was amplified from the coding region of housekeeping gene *ddlA*, which does not share sequence identity with the intergenic region upstream of *bsrX*. EMSAs in the presence of 200-fold unlabeled specific probe (S, upstream of *lplA*) or non-specific (NS, negative control) competitor DNA were performed as controls. Free DNA fragments and BsrR-DNA complexes are labeled.

Expression of 25 putative target genes was measured by RT-qPCR, while expression of *HMPREF035_10301* and *10302* was not measured due to lack of suitable specific primers. Among 25 tested genes, 16 genes were differentially expressed between the *E. faecium* NW2 wild-type and the *bsrR* mutant after exposure to 0.5% ox gall (Figure 5). Three genes, *bsrX*, *bsrR*, and *bsrS*, exist in an operon *bsrXRS* as described above. *HMPREF0351_12077* is annotated as encoding a lipoate-protein ligase A (*lplA*). The *lplA*, *12078* and *12079* were co-transcribed and belong to a same operon (Figures 6A,B). As shown in Figure 6C, BsrR was able to bind the upstream of *lplA* gene, indicating that BsrR directly regulate the *lplA* operon. Seven genes are putatively involved in amino acid metabolism (*HMPREF0351_11989* and *10965*), fatty acid biosynthesis (*HMPREF0351_11295*), secondary metabolites biosynthesis (*HMPREF0351_10954*), inorganic ion transport and metabolism (*HMPREF0351_10955*), cell wall maintenance (*HMPREF0351_10944*), and DNA mismatch repair (*HMPREF0351_11678*). The other target genes encode hypothetical proteins of unknown functions (*HMPREF0351_11172*, *11173*, *12076*, and *12672*).

## Discussion

Bile salts execute antimicrobial effects through disturbing cell membrane and macromolecule stability, and inducing oxidative stress, DNA damage and protein mis-folding in bacterial cells (Ruiz et al., 2013). Thus, the ability to adapt and respond to bile salts is essential for the survival and persistence of intestinal bacteria in gastrointestinal tracts. Two-component systems (TCSs) are dominant regulated mechanisms of bacteria in response to environmental stimuli (Groisman, 2016). Several studies have shown that TCSs are involved in resistance to bile salts in various bacterial species. However, the involvement of TCSs in *E. faecium’*s resistance to bile salts has not been reported. As a successful colonizer in the gastrointestinal tract of various hosts including humans and animals, *E. faecium* must have evolved mechanisms to sense, respond to and resist bile salts. The results from the current study indicate that LiaFSR and BsrXRS contributed to bile salt resistance of *E. faecium*.

LiaFSR was shown to be involved in bile resistance in *E. faecium* isolates. The positive correlation between the survival rate in 0.5% ox gall and the expressions of *liaSR* genes tended to be significant. More importantly, disruption of *liaR* or *liaS* significantly reduced the survival rate of mutants in different concentrations of bile salts compared to the *E. faecium* wild type. Our results for the LiaFSR system in *E. faecium* were supported by a previous study in *E. faecalis* in which the *liaR* mutant had a low survival rate after exposure to bile (Harp et al., 2016). The LiaFSR system was first identified in the response against lipid II interfering antibiotics (bacitracin, nisin, ramoplanin, and vancomycin) in *Bacillus subtilis* (Mascher et al., 2004). The role of LiaFSR in *E. faecium* and *E. faecalis* has mainly been described recently in the context of daptomycin resistance (Reyes et al., 2015). Given that cell membrane is a major attack target of bile salts, our finding expands the role of LiaFSR in dealing with cell membrane stress in *E. faecium*.

Cross-resistance against bile salts and antibiotics may exist. Saito et al. (2014) found the supplementing cultures with 0.2% bile significantly enhanced survival of *E. faecalis* OG1RF when exposed to daptomycin through incorporation of exogenous fatty acids and altering the fatty acid composition of cell membrane. In addition, LiaR-deficient strain of *E. faecalis* can incorporate exogenous fatty acids into its membrane, then increase the survival rate when exposed to a variety of membrane damage agents, such bile, SDS and daptomycin (Harp et al., 2016).

The functions of BsrXRS have not been explored before. In this study, expression of *bsrR* and *bsrS* was significantly correlated with the survival rate of *E. faecium* in 0.5% ox gall. There were 4 amino acid polymorphisms (F for NW1, 2, 3, 5, and 6 and V for NW4, 7, and 8 at position 176; K for NW2, 3, 4, 7, and 8 and R for NW 1, 5, and 6 at position 243; A for NW1, 2, 3, 5, and 6 and T for NW4, 7, and 8 at position 471; K for NW1, 2, 3, 5, and 6 and E for NW4, 7, and 8 at position 481) in the BsrS protein, while no difference was found in the BsrR protein. The amino acid at position 176 is located in a predicted *trans*-membrane domain in the sensor histidine kinase BsrS, which is essential for signal transmission from extracellular to cytoplasm (Zschiedrich et al., 2016). The other two amino acid substitutions at positions 471 and 481 are located in C-terminal ATP-binding catalytic domain, which is responsible for autophosphorylation of HK. These amino acid substitutions may affect the signal transduction and activation of BsrR and change the transcription of target genes, resulting in different bile salt resistance in *E. faecium* strains. The sequence analysis of BsrX using revealed that BsrX harbors 3 transmembrane helices, suggesting BsrX may be a small membrane protein (116 aa). Despite repeated attempts, we were unable to obtain a mutant strain of *bsrX* gene. Therefore, the function of *bsrX* gene was not validated in the present study.

Twenty-seven potential target genes for BsrR were revealed and these target genes are involved in multiple cellular functions. Sixteen genes were found as differentially expressed between *E. faecium* NW2 wild-type and *bsrR* mutant after exposure to 0.5% ox gall. Of the 16 differentially expressed genes, 7 genes (*bsrX*, *bsrR*, *bsrS*, *lplA*, *HMPREF0351_10944*, *11173*, *12672*) were upregulated after exposure to 0.5% ox gall. Our result was supported by a previous study in *E. faecium* E1162 in which these 7 genes were also upregulated after addition of bile salts to the culture medium (Zhang et al., 2013). However, none of the 7 genes showed significant differential expression in *E. faecalis* V583 in the presence of ox gall (Solheim et al., 2007). On the other hand, a TCS CroRS system was induced in *E. faecalis* V583 after exposure to ox gall (Solheim et al., 2007). However, expression of the CroRS system was not significant in *E. faecium* NW2 in our study and also in *E. faecium* E1162 (Zhang et al., 2013). Although *E. faecium* is a close relative of *E. faecalis*, these findings imply that different mechanisms are involved in bile resistance in *E. faecium* and *E. faecalis*.

Among these 16 differentially expressed target genes of BsrR, 3 genes (*bsrX*, *bsrR*, and *bsrS*) constitute an operon *bsrXRS* as discussed above. Another operon targeted by BsrR consists of 3 genes, *lplA* encoding a lipoate–protein ligase A (*HMPREF0351_12077)*, a gene encoding HD family metal-dependent phosphohydrolase (*HMPREF0351_12078)* and a gene encoding HAD superfamily hydrolase (*HMPREF0351_12079*). Whether involvement of the HD family metal-dependent phosphohydrolase and HAD superfamily hydrolase in bile resistance is unknown, *lplA* gene may play an important role in bile salt resistance. This was supported by the observation that mutation of *lplA* induced a 2-log reduction of viability in comparison with the wild type *L. monocytogenes* after 8 h exposure to porcine gallbladder bile (Dowd et al., 2011). LplA catalyzes the formation of lipoyl-AMP from lipoate and ATP and transfers the lipoyl moiety to a specific lysine residue on the acyltransferase subunit of α-ketoacid dehydrogenase complexes. In particular, the branched-chain-ketoacid dehydrogenase (BCDH) participates in the degradation of branched chain amino acids to generate branched-chain CoA (BC-CoA) molecules. In many Gram-positive bacteria, these short BC-CoA molecules are used chiefly as primers for generating longer branched-chain fatty acids that can modulate membrane fluidity. This change of membrane fluidity may promote *E. faecium* to resist bile salts toxicity. However, further study is required to corroborate this hypothesis.

Bacterial cell membrane and cell wall are main targets of bile salts. Thus changes in their composition appear to be the most prevalent actions to resist bile salt in bacteria (Ruiz et al., 2013). Studies have shown that resistance to bile salt in *E. faecalis* involved changes in fatty acid biosynthesis (Solheim et al., 2007) and in membrane composition or cell wall synthesis (Rincé et al., 2003). In addition, DNA mismatch repair proteins repair the DNA damage caused by bile salts and are important for bacterial bile resistance (Rincé et al., 2003). In this study, the target genes of BsrR from functional annotation are involved in amino acid metabolism (*HMPREF0351_11989* and *10965*), fatty acid biosynthesis (*HMPREF0351_11295*), secondary metabolites biosynthesis (*HMPREF0351_10954*), inorganic ion transport and metabolism (*HMPREF0351_10955*), cell wall maintenance (*HMPREF0351_10944*), and DNA mismatch repair (*HMPREF0351_11678*). These target genes may contribute to bile salt resistance in *E. faecium*.

The *Enterococcus* genus is an ancient genus of microbes that are highly adapted to living in complex environments and surviving harsh conditions. The BsrXRS system is highly conserved in *Enterococcus* genus, indicating that BsrXRS may play a prominent role in responding to environmental changes among bacteria in the *Enterococcus* genus.

## Conclusion

In summary, our data demonstrate that *E. faecium* can improve the resistance to bile salts by up-regulating LiaFSR and BsrXRS systems. The BsrXRS system up-regulates gene expression of target genes that involving in change of membrane fluidity, fatty acid biosynthesis, cell wall maintenance, DNA mismatch repair. These findings provide potential molecular mechanisms for resistance to bile salts by *E. faecium*.

## Author Contributions

LZ and XZ contributed to the design of the experiments and prepared the manuscript of this publication. LZ, LW, PT, and TB performed the experiments. LZ and LL analyzed the data. All authors read and approved the final manuscript.

## Conflict of Interest Statement

The authors declare that the research was conducted in the absence of any commercial or financial relationships that could be construed as a potential conflict of interest.
