# Supplementary material for: The LiaFSR and BsrXRS Systems Contribute to Bile Salt Resistance in Enterococcus faecium Isolates
Source: Front Microbiol. 2019 May 10;10:1048. doi: 10.3389/fmicb.2019.01048 (PMC6522849; doi:10.3389/fmicb.2019.01048)
Supplement: Supplementary file 1 [file Data_Sheet_1.docx]

***Supplementary Material***

**Supplementary Tables**

**Supplementary Table S1.** Primers used in this study

| Name | Locus tag | Sequence (5’-3’) |
| --- | --- | --- |
| **16S rRNA gene** |  |  |
| 27F |  | AGAGTTTGATCCTGGCTCAG |
| 1492R |  | GGTTACCTTGTTACGACTT |
| **RT–qPCR** |  |  |
| RR1-q-F | HMPREF0351_10436 | AGTTGCTTTACCGTTCTGTGTTG |
| RR1-q-R |  | GGTGTGTCAAATCTGGTGCTAC |
| HK1-q-F | HMPREF0351_10437 | TTTTGTTGGGCGGCGTATCC |
| HK1-q-R |  | TCTGCTACTTTCTGCTGTTCATCC |
| RR2-q-F | HMPREF0351_12644 | CCACACCATTCACATTTACATCCC |
| RR2-q-R |  | TCGGCATCCAACTTCGTCATC |
| HK2-q-F | HMPREF0351_12643 | GACCACCATATTCTTGTCGTTCAG |
| HK2-q-R |  | GGCTGTTGCTTCTCGTTGTTATC |
| RR3-q-F | HMPREF0351_11400 | AGAACAAGTTTTGCCCATACAAGG |
| RR3-q-R |  | AATCGTCGTATCAGAATCAAATCCC |
| HK3-q-F | HMPREF0351_11399 | CGAAAGAGACTGGCGGATACG |
| HK3-q-R |  | CGACTCCTTGTGGAAGATAATTGG |
| RR4 (BsrR)-q-F | HMPREF0351_11748 | CCATCTCTAAGCCGATCACCTG |
| RR4 (BsrR)-q-R |  | AGACCTGAATTTGCCCACACTG |
| HK4 (BsrS)-q-F | HMPREF0351_11747 | TTTCATTCACCAAACGGATCAGAC |
| HK4 (BsrS)-q-R |  | GAACACCTCTTACGACAATCAACG |
| RR5-q-F | HMPREF0351_11843 | AACCTTCTATTGTAACGCTCTTGAC |
| RR5-q-R |  | ATTCTAATCCTTCGCCTCCATCTG |
| HK5-q-F | HMPREF0351_11844 | CCTGATTGATGATGCTGATTCTCG |
| HK5-q-R |  | TGACTTCTGTGTACCGCTGTTC |
| ChtR-q-F | HMPREF0351_10536 | CTTCGCCGTTCTTACAACTACAC |
| ChtR-q-R |  | CTCTCCGTTGATTTCCATACTTCC |
| ChtS-q-F | HMPREF0351_10537 | AGTGGTGGGAGGACAGAAGAAG |
| ChtS-q-R |  | CTTGTTGATTACTGATTGCTGATTGC |
| CroR-q-F | HMPREF0351_12687 | CTTGCTTGCTAGTCATCCTAATCG |
| CroR-q-R |  | TTTCACCACCAGTTGCTTCTTC |
| CroS-q-F | HMPREF0351_12688 | CGTATCGGTTCAGAAGTTGTTGC |
| CroS-q-R |  | AAGTCCAGTTCCTCCAGTTGC |
| LiaR-q-F | HMPREF0351_10938 | GTCATGGAAGAAATGGATGGTATCG |
| LiaR-q-R |  | CGTTGCGTTGCCCTTATAGC |
| LiaS-q-F | HMPREF0351_10937 | GGCATTGACGGAGCAAGCAG |
| LiaS-q-R |  | CGCATCTCAGACTGGGAAGC |
| BsrX-q-F | HMPREF0351_11749 | GCCGATCAATGCCATCAGTAAG |
| BsrX-q-R |  | TGGGTATCATTCGCCTCTTGC |
| 11750-q-F | HMPREF0351_11750 | TCTCCCCGTCATCGCTACAG |
| 11750-q-R |  | AGATTTCTTCGCCGTCTACTCG |
| 12076-q-F | HMPREF0351_12076 | GGAGAAATGCGGATGCGTTTG |
| 12076-q-R |  | CTGCTGTGGGACGGTCTTTC |
| 12077-q-F | HMPREF0351_12077 | CAGGTGGATTAGGGATCGTATCAG |
| 12077-q-R |  | TGCTTGTATCGGTGCCTTTGG |
| 12078-q-F | HMPREF0351_12078 | ACAAGGCAGACATCGCACAC |
| 12078-q-R |  | CACGCAAGGAGGGATCAACC |
| 12079-q-F | HMPREF0351_12079 | GTCGGCAGTCATCTCCATTCG |
| 12079-q-R |  | GGTGTCATTTCTTCAGGTGTTCG |
| 11989-q-F | HMPREF0351_11989 | TCGTCCACCGTAGAGATGAATTAC |
| 11989-q-R |  | CCTGTCACTACCATTTCGTTTCC |
| 12122-q-F | HMPREF0351_12122 | ACAAGCGGAGTCCTATGAATCG |
| 12122-q-R |  | TCTACGCCAGTCATCAAAGCC |
| 10965-q-F | HMPREF0351_10965 | CGATGACAGGAATGACGGAAATG |
| 10965-q-R |  | ACGAGGAGAATAATTGCTTAAAGACC |
| 11678-q-F | HMPREF0351_11678 | GGCTCTTCGTGTCGCTTATTTC |
| 11678-q-R |  | CTTTGTCCGTGATTTCTTTCATTGC |
| 10295-q-F | HMPREF0351_10295 | CGGAACAATGAACGGACTATGC |
| 10295-q-R |  | TCTTCTCTACTGCTGCTACTTTGG |
| 10296-q-F | HMPREF0351_10296 | CAGTAGCAATCGGAATGGCAATC |
| 10296-q-R |  | ATTAGGCATCATCATCTTCATAGCG |
| 11918-q-F | HMPREF0351_11918 | TGGCAGTAGAATTGGCACAGTC |
| 11918-q-R |  | TGAGCAGATAGAGCCGTTGAATC |
| 10954-q-F | HMPREF0351_10954 | AGCCCATTGTATCCACTTCGTC |
| 10954-q-R |  | CTGTATTCAGAAAGAATCGCCATCC |
| 10955-q-F | HMPREF0351_10955 | TGCGGGATTGACAAACCTAATTC |
| 10955-q-R |  | CACAACCACACAACAAAGTAATGC |
| 11172-q-F | HMPREF0351_11172 | CGAATAAACTGCCAAAGACATACAATC |
| 11172-q-R |  | AAGAAGCCTCGCCTTTCACAC |
| 11173-q-F | HMPREF0351_11173 | TTGTTACTAATAGGGATTTGGGGTATC |
| 11173-q-R |  | AACTCCATGTCTTATAATGTTCTATCG |
| 11295-q-F | HMPREF0351_11295 | GAAACAAGGGGCAGAAATTATCTATAC |
| 11295-q-R |  | CAGCATATTCACCGATGGCATTC |
| 11296-q-F | HMPREF0351_11296 | ACACTGGCACAAGCAGGTTC |
| 11296-q-R |  | GCTGTACCGACTGATCCCTTC |
| 12672-q-F | HMPREF0351_12672 | CTTGGCTGGCTTGGCATACC |
| 12672-q-R |  | TTCGTTATCTGCTATCGTCATCGG |
| 12673-q-F | HMPREF0351_12673 | ACCAAGACGGAACGATTATAGGAAC |
| 12673-q-R |  | GTCGCTTGAAGTTGATGAATCTGAG |
| 11330-q-F | HMPREF0351_11330 | GGTGTAGGAGCAGGAGCAGTC |
| 11330-q-R |  | GCAGTTGAATCCAGATTGAAAGGC |
| 10944-q-F | HMPREF0351_10944 | TGAATGCGGTAAGTTCTTCTTCTAC |
| 10944-q-F |  | GCAAGGATAACAATAATTTGAGTGATG |
| adk-q-F | HMPREF0351_10079 | GGTATTCCGCACATCTCAACAG |
| adk-q-R |  | CGCTCTTTCACGATTCCATTTG |
| **RT-PCR** |  |  |
| P1 | HMPREF0351_11749 | aaataaaactccttttttattggaat |
| P2 | HMPREF0351_11748 | CCATCTCTAAGCCGATCACCTG |
| P3 | HMPREF0351_11748 | gaaaacacgagaagcgtatctg |
| P4 | HMPREF0351_11747 | tgttctgtcagtacttgctctg |
| P5 | HMPREF0351_12077 | tcaacggtaatcaggaaaaacg |
| P6 | HMPREF0351_12078 | atgaatgggtaaaacggctatg |
| P7 | HMPREF0351_12078 | ccttatgatttttatcgcccag |
| P8 | HMPREF0351_12079 | ctaggaaatcttcatgagtcag |
| **Gene mutation** |  |  |
| M13-F |  | TGTAAAACGACGGCCAGT |
| SK-R |  | CGCTCTAGAACTAGTGGATC |
| LiaR-mut-F |  | TTGGGTACCgcttaggcgtctcttcttattt |
| LiaR-mut-R |  | CAGGAATTCgtgtaataaacaattcgtccgc |
| LiaS-mut-F |  | TTGGGTACCTAACCAAAGTCATTGGTGGGGA |
| LiaS-mut-R |  | CAGGAATTCTATTAAATCCTGTCCCGTCGTC |
| BsrR-mut-F |  | TTGGGTACCacccacaacattgggatatga |
| BsrR-mut-R |  | CAGGAATTCcgtaatttcttgatatgggcgt |
| BsrS-mut-F |  | TTGGGTACCCAGAGCAAGTACTGACAGAACA |
| BsrS-mut-R |  | gacCTCGAGGACATTCCAATTCCCGTATCTTC |
| LiaR-d-check |  | acgggaagttatggattgaac |
| LiaS-d-check |  | GATGAAAATCCTCGCAGATTGAAG |
| BsrR-d-check |  | tttaaagacatctgggcatacg |
| BsrS-d-check |  | TCGATGATTCTGGTGTTGCC |
| **Gene**  **Complementation** |  |  |
| LiaS-comp-F |  | cgGGTaccCGTCAAAGTAGAACAAGCGTA |
| LiaS-comp-R |  | cgggatccCTATGCCTCCTTCATCAAAGG |
| **EMSA** |  |  |
| BsrRc-F |  | CGGGATCCATGAATATTTTAATGATTGAAGATAATC |
| BsrRc-R |  | TGCTCGAGTTAGGCAACACCAGAATCATCGA |
| pBsrRS-F |  | 5’-biotin-tctttccctcctaaattctcttc |
| pBsrRS-R |  | aaataaaactccttttttattggaat |
| pLplA-F |  | 5’-biotin-tattcattctcctttacttctcatagc |
| pLplA-R |  | tcaaccggctcctttgtataaa |
| pNC-F |  | 5’-biotin-CTTTGCTTTATCCGATGTTTGTC |
| pNC-R |  | GCTTCAATTCCTTGTTCAACG |

**Supplementary Table S2.** The TCSs that were studied for possible involvement in bile salts resistance in *E. faecium* isolates

| Gene  in our study | Locus-tag *in E. faecium* DO (accession no.) | Description |
| --- | --- | --- |
| HK1 | HMPREF0351_10437  (YP_006375043.1) | 36% identity to BaeS of *L. rhamnosus GG* (CAR86050.1) |
| RR1 | HMPREF0351_10436  (YP_006375042.1) | 43% identity to BaeR of *L. rhamnosus GG* (CAR86051.1) |
| RR2 | HMPREF0351_12644  (YP_006377250.1) | 36% identity to CpxR of *Klebsiella pneumoniae* NTUH-K2044 (BAH60995.1) |
| HK2 | HMPREF0351_12643  (YP_006377249.1) | 25% identity to CpxA of *Klebsiella pneumoniae* NTUH-K2044 (BAH60994.1) |
| RR3 | HMPREF0351_11400  (YP_006376006.1) | 27% identity to HK of *L. acidophilus NCFM* (AAV43255.1) |
| HK3 | HMPREF0351_11399  (YP_006376005.1) | 39% identity to RR of *L. acidophilus NCFM* (AAV43256.1) |
| RR4  (BsrR) | HMPREF0351_11748  (YP_006376354.1) | 73% identity to PhoP3 of *L. rhamnosus GG* (CAR86898.1) |
| HK4  (BsrS) | HMPREF0351_11747  (YP_006376353.1) | 49% identity to PhoR3 of *L. rhamnosus GG* (CAR86899.1) |
| RR5 | HMPREF0351_11843  (YP_006376449.1) | 40% identity to BL1000 of *Bifidobacterium longum* NCIMB 8809 (AAN24808.1) |
| HK5 | HMPREF0351_11844  (YP_006376450.1) | The cognate histidine kinase of RR5 |
| ChtR | HMPREF0351_10536  (YP_006375142.1) | 100% identity to ChtR of *E. faecium* E1162 (EFF34003.1), which contributes to chlorhexidine tolerance |
| ChtS | HMPREF0351_10537  (YP_006375143.1) | 100% identity to ChtS of *E. faecium* E1162 (EFF34002.1) |
| CroR | HMPREF0351_12687  (YP_006377293.1) | 100% identity to CroR of *E. faecium* 1,141,73 (EEV51100.1), which responds to cell wall-targeting antibiotics in *E. faecium.* |
| CroS | HMPREF0351_12688  (YP_006377294.1) | 99% identity to CroS of *E. faecium* 1,141,733 ( EEV51099.1) |
| LiaR | HMPREF0351_10938  (YP_006375544.1) | 73% identity to VraR of *L. rhamnosus GG* (CAR87605.1), which resistant to cell membrane-targeting lipopeptide daptomycin. |
| LiaS | HMPREF0351_10937  ( YP_006375543.1) | 49% identity to VraS of *L. rhamnosus GG* (CAR87606.1) |

**Supplementary Tables and Figures**


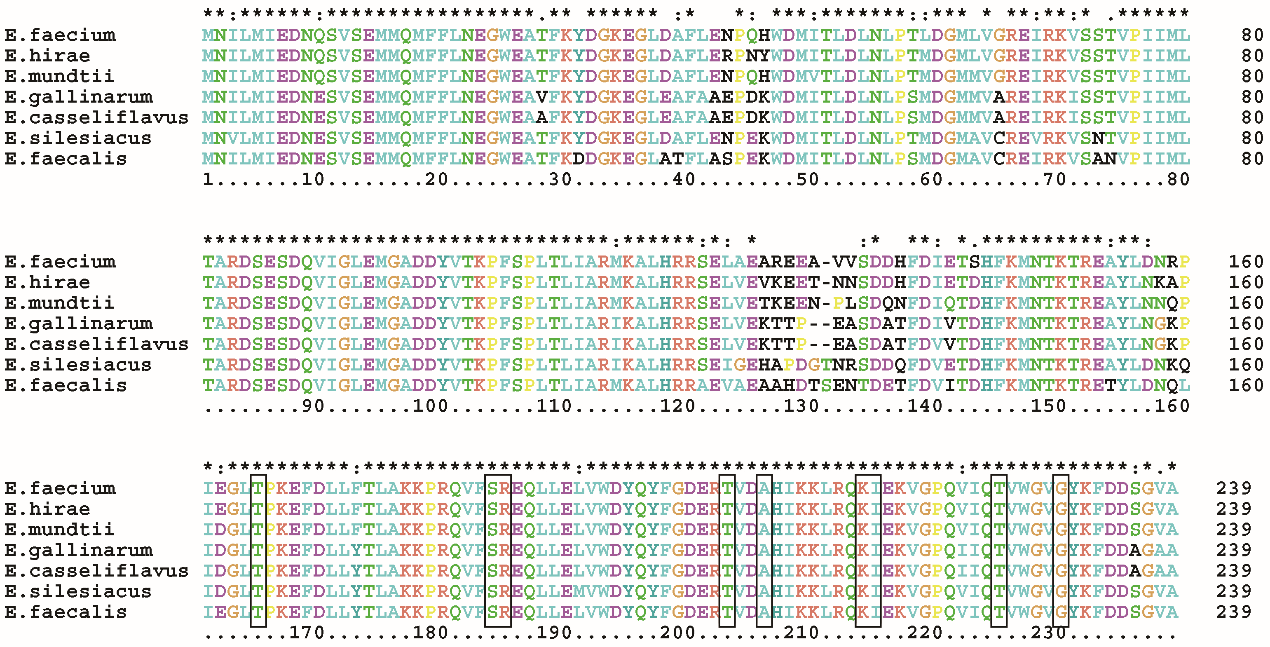


**Supplementary Figure S1.** Sequence analysis of the BsrR protein from various bacteria. Multiple alignment of the amino acid sequences of BsrR from various bacteria using Clustal W. Identical amino acids are marked by an asterisk (*), and conserved (:) and semiconserved substitutions (.) are marked by two dots and by a single dot, respectively. Nine proposed DNA-binding site (Thr-164, Ser-183, Arg-184, Thr-202, Ala-205, Lys-213, Ile-214, Thr-224, Gly-229) are marked by red box. The following BsrR orthologs were used to multiple alignment: *E. faecium* (*Enterococcus faecium* DO, GenBank accession number YP_006376354); *E. hirae* (*Enterococcus hirae* ATCC 9790, GenBank accession number AFM70844); *E. mundtii* (*Enterococcus mundtii* QU 25, GenBank accession number BAO07335); *E. gallinarum* (*Enterococcus gallinarum* FDAARGOS_163, GenBank accession number AMG50040); *E. casseliflavus* (*Enterococcus casseliflavus* EC20, GenBank accession number EEV40649); *E. silesiacus* (*Enterococcus* *silesiacus* LMG 23085, GenBank accession number ALS00538); *E. faecalis* (*Enterococcus faecalis* V583, GenBank accession number NP_814983).
